# MCM4 acts as a biomarker for LUAD prognosis

**DOI:** 10.1111/jcmm.17819

**Published:** 2023-10-10

**Authors:** Yue Tan, Lei Ding, Guiyuan Li

**Affiliations:** ^1^ Branch of Minhang, Department of Medical Oncology Fudan University Shanghai Cancer Center Shanghai China; ^2^ Department of Ultrasonic Diagnosis Second Affiliated Hospital of Anhui Medical University Hefei China; ^3^ Department of Oncology, School of Medicine, Tongji Hospital Tongji University Shanghai China

**Keywords:** cell cycle, lung adenocarcinoma, MCM4, overall survival, P21

## Abstract

MCM4 forms the pre‐replication complex (MCM2‐7) with five other minichromosome maintenance (MCM) proteins. This complex binds to replication origins at G1 stage in cell cycle process, playing a critical role in DNA replication initiation. Recently, MCM4 is reported to have a complex interaction with multiple cancer progression, including gastric, ovarian and cervical cancer. Here, this study mainly focused on the expression of MCM4 and its values in lung adenocarcinoma (LUAD). MCM4 was highly expressed in LUAD tumours and cells, and had an important effect on the overall survival. Overexpression of MCM4 promoted the proliferation, and suppressed the apoptosis in LUAD cells. However, MCM4 silence led to the opposite results. In vivo, knockdown of MCM4 inhibited tumour volume and weight in xenograft mouse model. As a member of DNA helicase, knockdown of MCM4 caused cell cycle arrest at G1 stage through inducing the expression of P21, a CDK inhibitor. These findings indicate that MCM4 may be a possible new therapeutic target for LUAD in the future.

## 1 INTRODUCTION

Cancer is characterized by high heterogeneity and complexity, and is related to a series of genetic and epigenetic aberrations.^1^, ^2^ Lung cancer has the highest incidence and is the biggest cause of cancer mortality worldwide. It consists of two main subtypes, including small cell lung cancer (SCLC) and non‐SCLC (NSCLC).^3^ The latter is the common form of lung cancers, making up approximately 85%, and it is further classified into three types: lung adenocarcinoma (LUAD), lung squamous cell carcinoma (LUSC) and large cell carcinoma histologic subtypes.^4^, ^5^, ^6^ LUAD develops from type II alveolar epithelial cells,^7^, ^8^, ^9^ and constitutes unique lung cancer subtypes with mutational landscapes and distinct cellular.^8^ Due to the absence of effective screening programmes and clinical symptoms, most patients are at the advanced stage of disease when they are first diagnosed. However, this is not the best time for treatment, which is probably the most significant reason for the high death rate of lung cancer patients.^10^ In recent years, although the basic research and clinical diagnosis of lung cancer have made great progress, LUAD patients still have poor prognosis.^11^, ^12^, ^13^ According to statistics, the 5‐year survival rate for NSCLC is less than 15%.^3^, ^4^ Therefore, it is high time to research on the molecular mechanisms underlying lung cancer development to determine useful diagnostic markers and more effective treatments.

The minichromosome maintenance (MCM) proteins are generated from MCM2‐7, which belong to AAA+ ATPase family.^14^, ^15^ MCMs interact with each other, and form a complex of a six‐membered replicative helicase.^16^ This complex plays a key role in binding to the replication origin, melting double‐stranded DNA (dsDNA) to initiate replication, and acting as a helicase on elongating DNA.^17^ During early G1 stage, MCM2‐7 complex is loaded on replication origins in a Cdt1‐ and Cdc18/Cdc18/Cdc6‐Dependent manner to form the pre‐replicative complex. Then, this complex unwraps the initiation DNA with the assistance of CDC45 protein and GINS complex, initiating DNA synthesis.^15^ In addition, MCM is reported to be one of the most valuable biomarkers for cancer diagnosis due to its abnormally high expression in tumour cells.^18^, ^19^

Among the subunits of MCM complex, MCM4 is considered as the most conservative protein in the entire evolutionary process.^15^ It is targeted to a region head‐to‐head a DNA‐activated protein kinase (PRKDC/DNA‐PK), which plays a significant role in DNA double‐strand breaks repair.^20^ MCM4 is a key to the initiation of eukaryotic genome replication, and has an important effect on replication forks formation and recruitment of other DNA replication‐related proteins.^21^ MCM4 high expression was observed in multiple cancers. Guo and colleagues^22^ found that MCM4 expression was closely correlated with tumour stage, and increased levels of MCM4 was related to better progression‐free survival (PFS) and overall survival (OS) in human gastric cancer. In laryngeal squamous cell carcinoma (LSCC), MCM4 suppression obviously caused cell proliferation inhibition and apoptosis induction. Furthermore, MCM4 overexpression was found in carcinoma tissues.^21^ Huang et al. found the positive rate of MCM4 was much higher in esophageal squamous cell cancer (ESCC) than in normal controls. Moreover, compared with stage T1 ESCC, MCM4 positive rate was significantly higher in stage T3 ESCC.^23^ Choy et al. claimed that MCM4 could act as prognostic factor in oesophageal carcinoma, ovarian and cervical cancer.^24^, ^25^ Kikuchi and colleagues also showed increased MCM4 level in NSCLCS cells. And, its abnormal expression was related to male gender, heavy smoking, poorer differentiation and non‐adenocarcinoma histology.^26^ However, the molecular mechanism underlying the relevance of MCM4 in NSCLC progression is still urgently needed to be studied.

Here, the present study researched MCM4 functions in LUAD development and explored its mechanism. High level of MCM4 was observed in LUAD tissues/cells in related to normal controls. And also, there was a clear correlation between MCM4 and OS. In addition, MCM4 overexpression promoted proliferation, suppressed apoptosis and accelerated cell cycle progression. While MCM4 silence resulted in the opposite results. What's more, MCM4 silence suppressed tumour growth in vivo.

## 2 MATERIALS AND METHODS

### 2.1 TCGA database analysis

MCM4 expression between tumour and normal tissues from LUAD patients was analysed. The expression data of MCM4 from 347 normal tissues and 483 tumour tissues were obtained from the TCGA database (https://portal.gdc.cancer.gov/). The data of 274 LUAD cases with low MCM4 expression and 274 cases with high MCM4 expression were obtained from TCGA database for OS analysis. Based on the multivariate logistic regression algorithm, the traditional prognostic model consisted of age and histological grade. Cox univariate and multivariate analyses were successively conducted to identify the independent prognostic factors.

### 2.2 Cell culture

Four human LUAD cell lines (H441, H460, H522, A549) and two human embryonic lung fibroblast cell lines (WI38 and MRC5) were obtained from the American Type Culture Collection (ATCC). These cells were cultured in RPMI 1640 medium (Invitrogen Life Technologies, Inc.) mixed with 10% fetal bovine serum (FBS, Gibco) and incubated at 37°C in a humidified atmosphere with 5% CO_2_.

### 2.3 Plasmid construction

To overexpress MCM4 in H441 and A549 cells, pcDNA3.1‐MCM4 expression plasmid was constructed. The inserted sequences were verified by DNA sequencing.

### 2.4 Cell transfection

Small interfering RNAs (siRNA) or short hairpin RNAs (shRNA) was used to inhibit MCM4 expression. The former was used for experiment in vitro, and the latter was used for constructing xenograft mouse model. Briefly, cells were seeded in six‐well plates overnight, and transfected with siMCM4 or shMCM4 or pcDNA3.1‐MCM4 mixed with lipofectamine 2000 solution (Invitrogen). The siRNA and shRNA targeting MCM4 (Target sequence:5′‐AAATGCATTCTTCAGCTATCCCTT‐3′) were bought from Santa Cruz Com.

### 2.5 Western blotting assay

To extract protein from cells, Ripa buffer with 50x protease inhibitor was used to lyses cells. We then used 10% sodium dodecyl sulfate (SDS) gel wells to isolate proteins at 80 V in the start and 120 V when samples reached into separating gel, and then transferred protein into nitrocellulose membranes (NC, Millipore). At the end of transfer, NC membranes were washed in PBS and incubated in 5% BSA to block protein at room temperature for 1 h. After incubated with primary antibodies overnight at 4°C, the membranes were washed by PBST following the incubation with secondary antibody for 1 h. Protein levels were evaluated by using infrared imaging system LI‐COR Odyssey. (Odyssey, LI‐COR). The following antibodies were used: anti‐MCM4 (#12973, 1:1000 dilution), anti‐PCNA (#13110, 1:1000 dilution), anti‐Caspase‐3 (#9662, 1:1000 dilution), anti‐Cleaved Caspase‐3 (#9664, 1:1000 dilution), anti‐P21 (#2947, 1:1000 dilution) and anti‐GAPDH (#5174, 1:10000 dilution) purchased from Cell Signalling Technology.

### 2.6 Colony formation assay

To detect clonogenic ability of a single cell, this study performed colony formation assay. After transfection, cells were cultured in six‐well plates at 1000 cells/well and cultured for 10 days. And then, we stained the cells for visualization by using 1% crystal violet. After washing and air drying, colonies pictures were taken. Colonies number in each well was counted.

### 2.7 MTT assay analysis

The present study detected cell viability by using MTT assay. After transfection, cells were seeded in 96‐well plates, and incubated with 5 mg/mL MTT for 3 h at 37°C. And then, the liquid supernatant was sucked and discarded. Each well was added with 150 μL DMSO. The OD value of each well at 450 nm was recorded by a microplate reader (Thermo Fisher Scientific). Graphs were plotted to show cell viability of different cells.

### 2.8 Cell apoptosis detection

To test cell apoptosis, this study examined the activity of caspase 3/7. After transfection, cells were cultured in 96‐well plates at 37°C with 5% CO_2_, and then collected, washed with PBS and lysed in lysis buffer. The pyrolysis productions are collected and centrifuged. The supernatant fluid mixed with reaction buffer as well as caspase 3/7 substrate, and incubated at 37°C for 4 h. In the end, the OD value at 405 nm was detected using a microplate reader (FACSCanto II system, BD).

### 2.9 Cell cycle detection

After transfection, cells were fixed with 70% ethanol overnight at 4°C. After washing, they were stained with PI and ribonuclease for 30 min at 37°C. After that, flow cytometer (FACSCanto II system, BD) was used to measure cell cycle phase.

### 2.10 Animal experiments

LSL‐Kras^G12D/+^ mice are LUAD models with a conditionally activatable allele of oncogenic K‐ras. This model is constructed by using a recombinant adenovirus, which express Cre recombinase (AdenoCre). The enzyme can express K‐ras G12D.^27^ Tumour tissues and adjacent normal tissue were collected for western blotting assay.

The animal experiments were carried following animal research guideline. Total 12 female Balb/c nude mice were used in this study, and divided into two groups. One group of mice was injected with A549 cells transfected with shMCM4 in the right flank. Another group including six mice was as controls injected with A549 cells. Tumour volume (mm^3^) was detected every 3 days by digital calliper, and calculated by the equation: 1/2 × length × width^2^. At the end of these experiments, the mice were scarified and tumours weight was measured.

### 2.11 Statistics analysis

Every experiment was performed three times individually in this study. All data was presented as the mean ± standard error. Statistically significant differences before and after treatment were calculated by Paired Student's *t*‐test. SPSS software was conducted to analyse all the statistics. *p* < 0.05 was identified as statistically significant difference.

## 3 RESULTS

### 3.1 MCM4 was upregulated in LUAD cells and tumour samples

**FIGURE 1:**
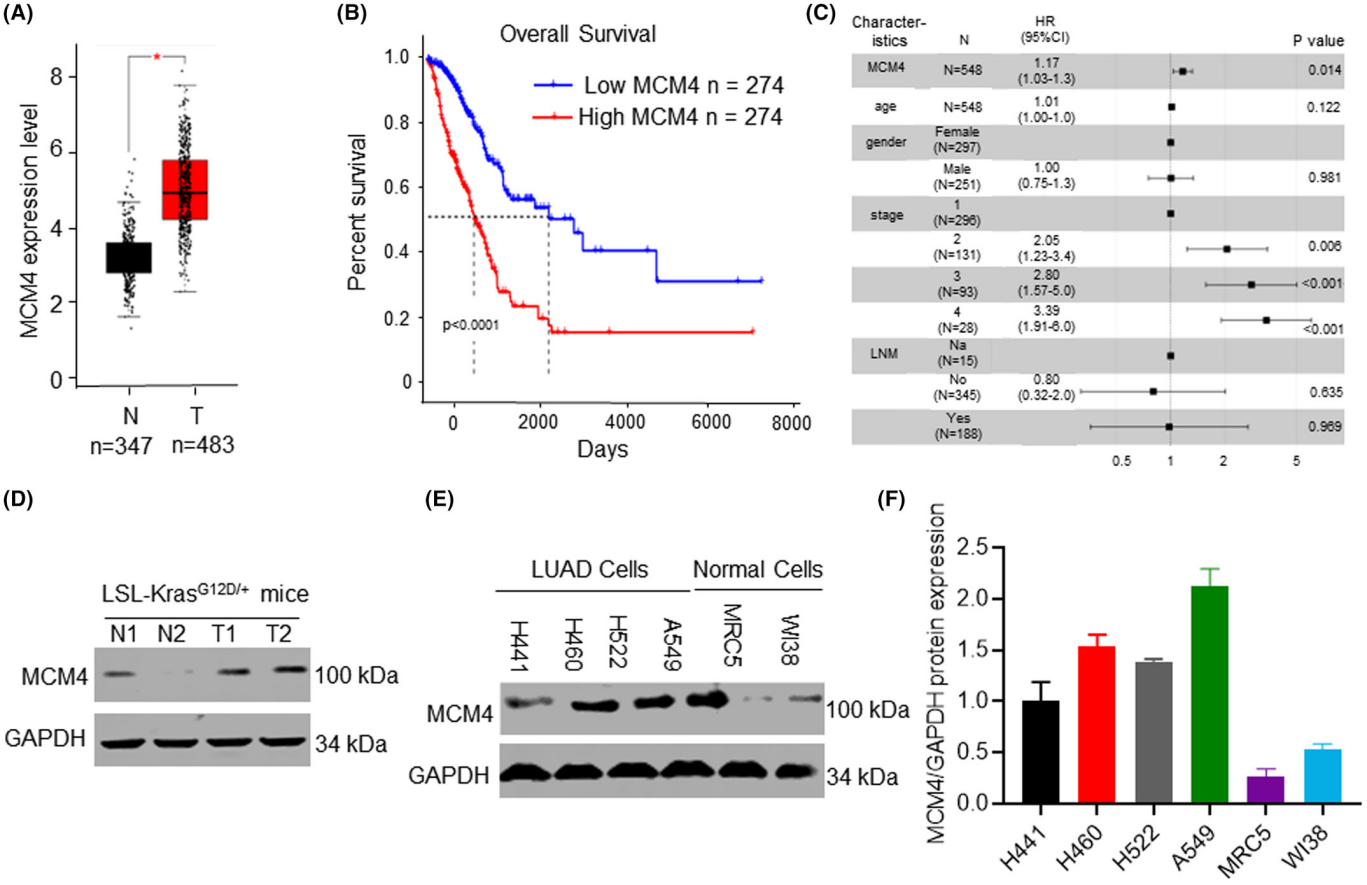
Elevated expression of MCM4 was observed in LUAD tissues and cells, which caused low overall survival rate. (A) MCM4 expression in TCGA datasets. MCM4 level in LUAD tumour samples was higher than that of adjacent normal tissues. (B) The overall survival rate of patients with high MCM4 expression was reduced compared with that with low MCM4 expression. (C) Cox univariate regression analysis of age and histological grade in TCGA cohort. (D) Western blotting assay showed MCM4 level was increased in tumour tissues in relative to those in normal tissues in LSL‐Kras^G12D/+^ mice model. (E) MCM4 was overexpressed in LUAD cells (H441, H460 and H552) compared with human embryonic lung fibroblast cells (MRC5 and WI38). *p < 0.05. N, adjacent normal tissues; T, tumour tissues. (F) Quantitative results of MCM4 expression.

To investigate the possible involvement of MCM4 in LUAD, our study first detected MCM4 expression in patients with LUAD, and evaluated the connection between its expression and OS by analysing TCGA database. A total of 483 tumour samples from LUAD patients and 347 normal samples were analysed. This study found high expression of MCM4 in tumour samples in relative to normal control groups (Figure 1A). Furthermore, significantly higher survival rate was observed in patients with downregulation of MCM4 than that of patients with upregulation of MCM4 (Figure 1B,C). LSL‐Kras^G12D/+^ mice models of LUAD showed the same results that increased levels of MCM4 were showed in LUAD samples in relative to control groups (Figure 1D). LSL‐Kras^G12D/+^ mice are LUAD models with a conditionally activatable allele of Kras.^27^ In addition, MCM4 levels in human embryonic lung fibroblast cells lines (MRC5/WI38) was significantly lower than that in LUAD cell lines (H441/H460/H522/A549) (Figure 1E,F). These findings revealed that MCM4 was upregulated in LUAD samples and cells, and its high expression was closely related to low survival rate.

### 3.2 MCM4 induced LUAD cell growth

**FIGURE 2:**
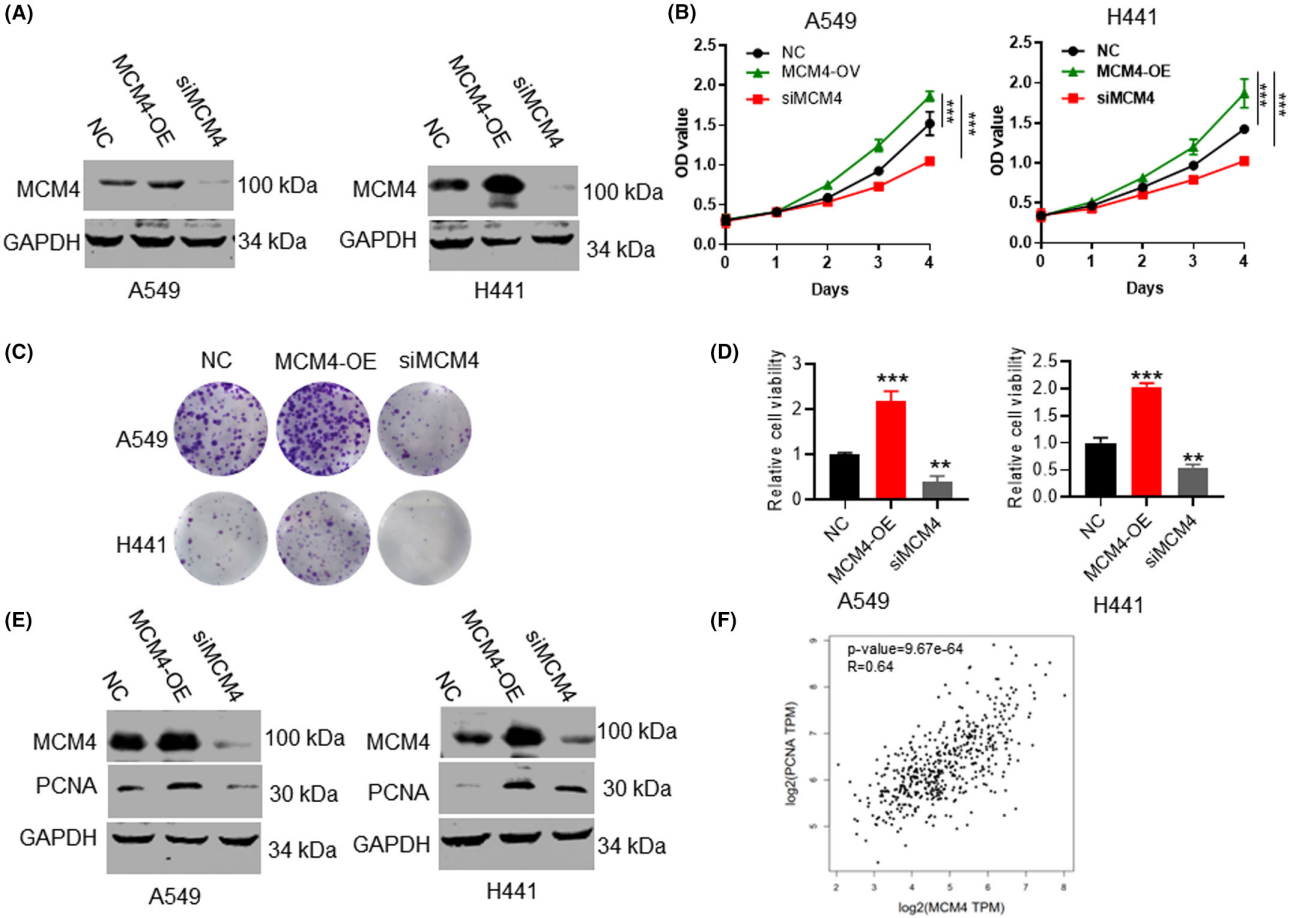
MCM4 promoted cell growth and viability in A549 and H441 cells. (A) Stable cell lines with MCM4 overexpression (MCM4‐OE) or knockdown (siMCM4) were constructed. MCM4 protein expression in these cells were validated using western blotting. (B) MTT assay analysis showed that MCM4 overexpression significantly increased the OD values of A549 and H441 cell lines, and MDM4 silence reduced the OD values. (C, D) Overexpression of MDM4 significantly increased colonies number of LUAD cells (A549 and H441 cell lines). On the contrary, the colonies number was decreased in cells with MCM4 knockdown. The relative number of colonies is calculated by normalization to untreated group as 100%. (E) PCNA is a biomarker for cell proliferation. MCM4 overexpression increased PCNA protein levels, whereas MCM4 inhibition reduced its level. Data are expressed as mean ± S.E.M. ***p < 0.001. MCM4‐OE, cells with MCM4 overexpression; NC, normal control; siMCM4, cells with MCM4 knockdown. (F) The expressive correlations between MCM4 and PCNA.

MCM4 was knockdown or overexpressed in A549 and H441 cells to detect the functions of MCM4 on LUAD cells growth. Its expression in these stable cells was verified by western blotting assay, suggesting the construction was successful (Figure 2A). MTT assay results suggested the absorbance of cells with MCM4 overexpression were more than that of normal control cell groups both in A549 and H441 cells, whereas cells with MCM4 knockdown had low OD values (Figure 2B). Additionally, this study carried on colony formation assay analysis. Overexpression of MCM4 in A549 and H441 cells led to increased colony numbers compared with normal cells, and MCM4 knockdown led to the opposite results (Figure 2C,D). PCNA, a marker for cell proliferation,^28^ was significantly upregulated in A549 and H441 cells caused by MCM4 overexpression (Figure 2E). While, inhibition of MCM4 suppressed PCNA protein levels. We also investigated the correlation between MCM4 and PCNA expression, and PCNA were positively correlated with MCM4 (Figure 2F). These findings revealed that MCM4 overexpression significantly enhanced the growth and proliferation of LUAD cell lines.

### 3.3 MCM4 inhibited LUAD cell apoptosis

**FIGURE 3:**
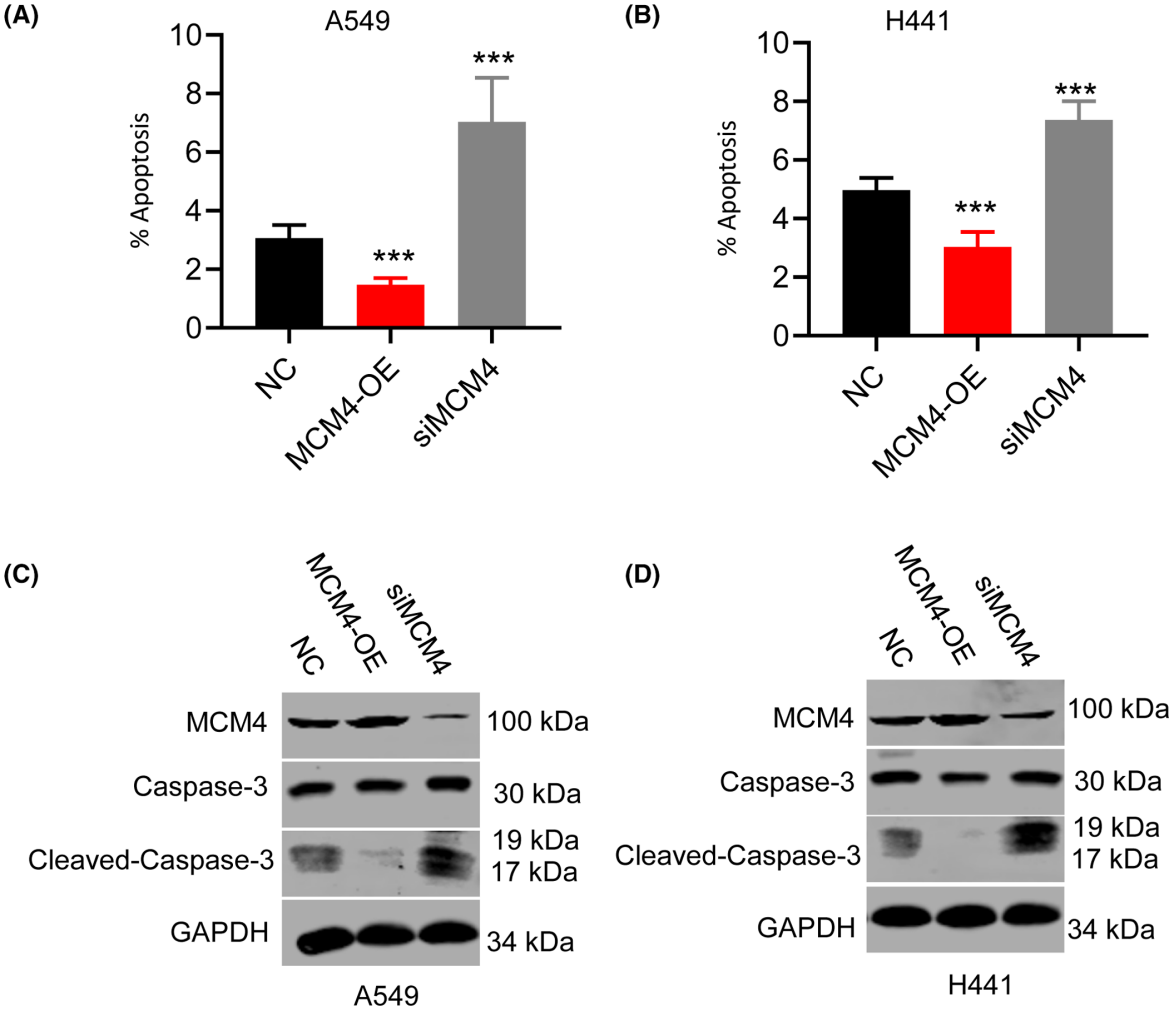
MCM4 suppressed apoptosis in LUAD cells. (A,B) Caspase 3/7 is a marker for apoptosis. Caspase 3/7 activity analysis showed that MCM4 overexpression significantly suppressed A549 (A) and H441 cells (B) apoptosis. While, MCM4 knockdown promoted cell apoptosis. (C,D) MCM4 overexpression inhibited cleaved capase‐3 protein levels, and MCM4 knockdown enhanced its levels in A549 (C) and H441 (D) cells. Caspase‐3 protein levels were mildly upregulated in H441 cells with MCM4 overexpression, and mildly downregulated in A549 cells with MCM4 knockdown. Data are expressed as mean ± S.E.M. ***p < 0.001. MCM4‐OE, cells with MCM4 overexpression; NC, normal control; siMCM4, cells with MCM4 knockdown.

Cell apoptosis was analysed in cells with MCM4 overexpression/silencing using caspase 3/7, a worthy marker for cell apoptosis. Upregulated MCM4 reduced caspase 3/7 activity in LUAD cells compared with control groups, while MCM4 knockdown caused the opposite results (Figure 3A,B). Caspase‐3 is recognized as critical enzyme in relation to apoptosis.^29^ Here we found overexpression of MDM4 could increase caspase‐3 protein expression, and decrease cleaved‐caspase‐3 protein levels (Figure 3C,D), and MDM4 knockdown led to the adverse results.

### 3.4 MDM4 accelerated cell cycle progression from G1 to S phase by suppressing P21 protein level

**FIGURE 4:**
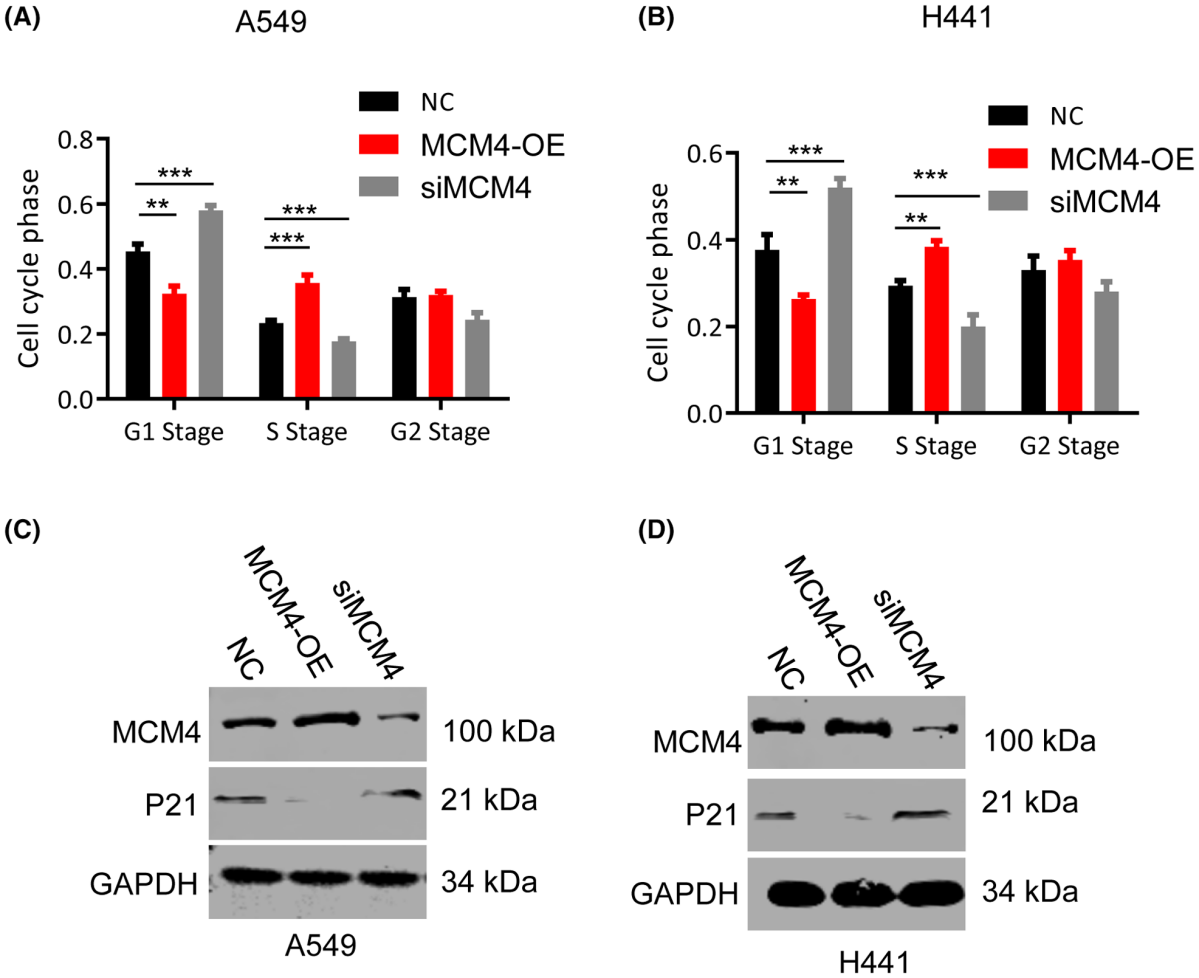
MCM4 was involved in cell cycle in LUAD cells. (A,B) Compared with normal cell lines, MCM4 overexpression accelerated cell cycle progression from G1 to S phase in both A549 (A) and H441 (B) cells. Whereas, MCM4 silence result in G1 arrest. (C, D). P21, an inhibitor of CDKs, induces G1 arrest and blocks the entry to S phase. Upregulation of MCM4 decreased protein level of P21, and MCM4 silence increased its level compared with control group. Data are expressed as mean ± S.E.M. **p < 0.01; ***p < 0.001. MCM4‐OE, cells with MCM4 overexpression; NC, normal control; siMCM4, cells with MCM4 knockdown.

Cell cycle phase in LUAD cells with MCM4 overexpression or knockdown was investigated using flow cytometry analysis. MCM4 upregulation accelerated cell cycle development from G1 to S phase, and MCM4 silence led to G1 phase arrest. Nevertheless, we did not find any significant difference at G2 phase (Figure 4A,B).

P21 was the well‐known tumour suppressor contributing to G1 stage arrest.^28^, ^30^, ^31^ In this study, western blotting assay showed that overexpression of MCM4 could inhibit P21 protein level and MCM4 silencing upregulated its level (Figure 4C,D). This may be the explanation for G1 stage arrest in cells with MCM4 knockdown.

### 3.5 Inhibition of MCM4 attenuated tumour growth in vivo

**FIGURE 5:**
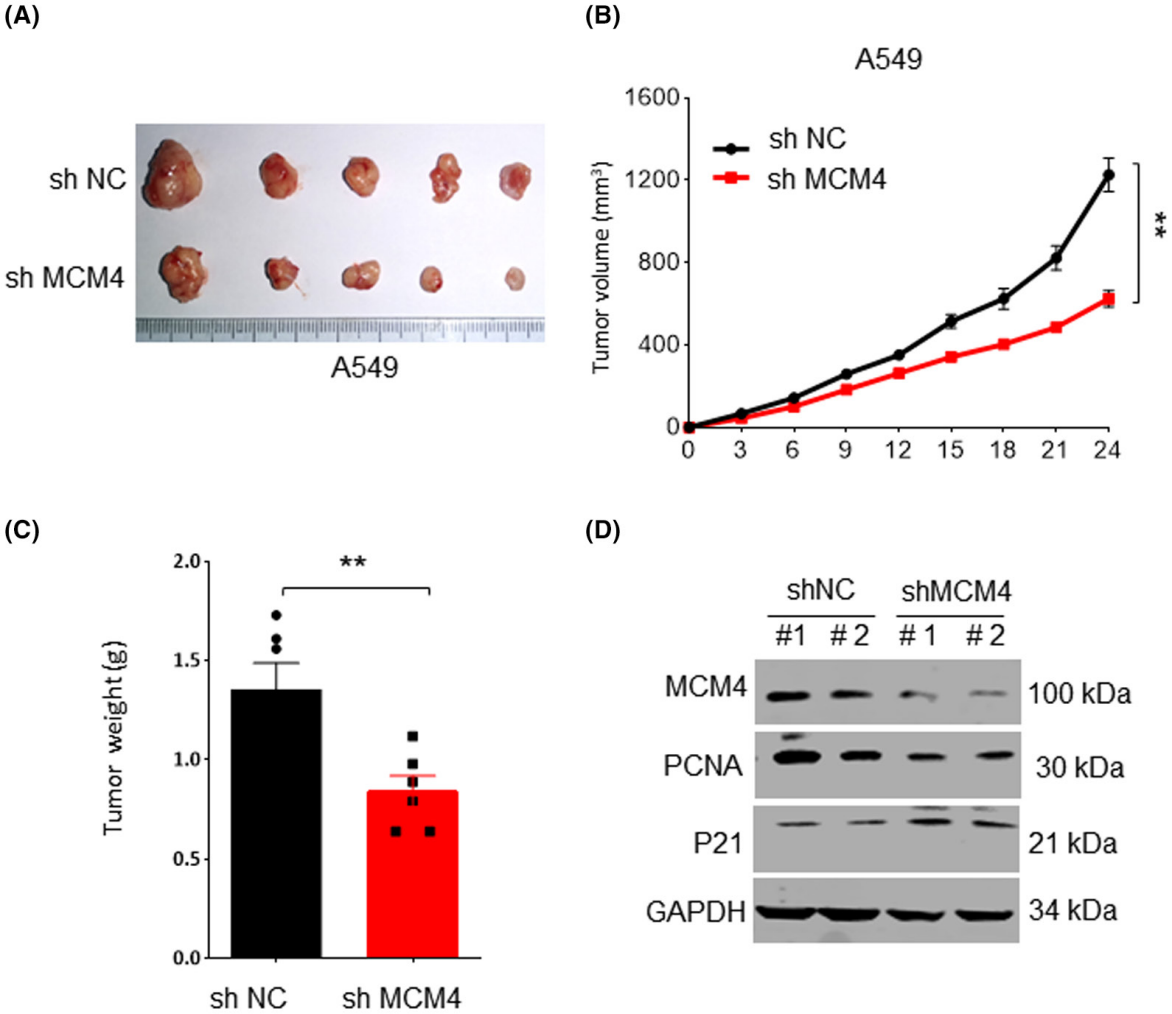
MCM4 silence inhibited tumour growth in vivo. (A) Female Balb/c nude mice were injected with A549 cells or cells with shMCM4. Tumour volume was measured every 3 days. After 24 days, tumours were removed and photographed. (B) MCM4 inhibition significantly decreased tumour volume compared with control group. (C) Tumour weight was weighed. MCM4 knockdown significantly inhibited tumour weight. (D) Consistent with the results of experiment in vitro, MCM4 silence also decreased PCNA and P21 protein levels. Data are expressed as mean ± S.E.M. **p < 0.01. sh NC, Mice injected with LUAD cells; shMCM4, Mice injected with LUAD cells with MCM4 knockdown.

The previous data we obtained in vitro suggested that MDM4 absence inhibited LUAD progression. We further evaluated the potential effects of MDM4 on tumour growth by using xenograft mouse model, which were injected with A549 cells or cells with MDM4 knockdown subcutaneously. The results presented in Figure 5 showed that MDM4 knockdown significantly decreased both tumour volume and weight after 24 days (Figure 5A–C). Here we also tested PCNA and P21 levels, and found that MCM4 silence inhibited its protein levels in tumour tissues (Figure 5D). PCNA is a protein that generated mainly in proliferating and transforming cells, and associated with DNA replication and replication‐related pathways.^32^ These findings suggested that MCM4 knockdown suppressed tumour growth via inhibiting PCNA levels.

## 4 DISCUSSION

MCM complex is composed of six subunits (MCM2‐7), playing a key role in DNA replication initiation.^15^, ^16^ Among MCM2‐7, MCM4 is considered as the most conservative protein, and its abnormal expression is associated with cancer progression, including mammary carcinoma, oesophageal and breast cancer.^18^, ^25^, ^33^ However, few reports focused on the role of MCM4 on lung cancer, especially LUAD. Therefore, this study investigated the impacts of MCM4 on LUAD development in vitro and in vivo. We found the oncogenic role of MCM4 in LUAD progression, suggesting that MCM4 could use as a reliable marker for LUAD diagnosis and treatment.

In details, we found MCM4 was overexpressed in LUAD tumour samples and cells in relative to their corresponding normal controls, and high MCM4 levels led to low survival rate in patients with LUAD. Previous research also reported excessive MCM4 expression in LSCC,^21^ ovarian,^34^ gastric,^22^ ESCC^23^ and breast cancer. MCM4 is high expression in breast cancer patient and silencing MCM4 significantly inhibited the proliferation of breast cancer cells. E2F2 induced upregulation of MCM4 expression in ovarian cancer, and was significantly associated with the poor prognosis of patients.^34^ In addition, excessive MCM4 expression is a potential prognostic marker for LSCC, which is related to the poor prognosis of patients.^21^ Those finding suggests that MCM4 may act as a pro‐oncogenic factor in most tumours, and may be involved in tumour formation and progression. Subsequently, this study found that MCM4 upregulation accelerated cell proliferation, and suppressed apoptosis in LUAD cells. While, MCM4 silencing caused the opposite results. In vivo, knockdown of MCM4 attenuated tumour growth in xenograft mouse model. Our results are similar to the findings of Han et al.^21^ and Junko et al.^26^ These results suggest that MCM4 is a potential molecular target for LUAD.

PCNA, a critical eukaryotic replication accessory factor, is highly conservative, and interacts with multiple proteins.^35^, ^36^ And it mediates DNA replication, apoptosis, repair and cell cycle control in vitro and in vivo. PCNA expression is reported to be unregulated in cancer cells and has been a biomarker for cell proliferation in tumours.^31^, ^37^ In this study, PCNA protein level was increased by MCM4 overexpression, and was decreased by MCM4 knockdown in A549 and H441 cell lines. The same results were observed in tumour tissues from mice models injected with LUAD cells with MCM4 knockdown. These findings further verified the carcinogenic role of MCM4 in LUAD.

Cell proliferation is mediated by multiple mechanisms. Uncontrolled self‐replication of tumour cells enhanced cancer development.^38^ MCM2‐7 is connected with DNA replication initiation, transcription, cycle checkpoint and RNA splicing.^39^ DNA replication, an important regulator, has the ability to precisely control cell cycle.^40^ Previous study has reported that the mutation of MCMs causes minichromosome instability in proliferating cells, obstructing cell cycle continuity.^39^ This study found that MCM4 overexpression promoted cell cycle progression from G1 to S phase, whereas MCM4 silence led to G1 phase arrest in LUAD cell lines. Consistent with our findings, Kikuchi et al.^26^ indicated that MCM4 silence caused G1 stage arrest in A549 cells; however, they found an opposite results in H1299 cells that MCM4 silence caused S phase arrest. Branzei et al.^41^ reported MCM2‐7 complex regulates cell cycle transition from G1 to S stage through controlling DNA replication checkpoints. Moreover, inhibition of MCM3 or MCM2, one of the other MCMs members, suppresses transition of G1 to S phase in colorectal cancer or lung cancer cells.^39^, ^42^ The complex acts as a DNA helicase, whose activation plays an important role in DNA synthesis initiation.^43^ In G1 stage, it is steadily loaded on chromatin, supplying helicase activity for double‐stranded DNA unwinding and DNA polymerase loading.^43^ The silence of MCM4 may result in the inactivation of MCM2‐7 helicase activity in G1 stage, affecting DNA synthesis, directly leading to G1 cell cycle arrest. Furthermore, this study found MCM4 overexpression inhibited the protein levels of P21, a key negative regulator of cell‐cycle progression. While, MCM4 inhibition increased P21 levels. P21, acting as the inhibitor of CDKs, could cause cell cycle arrest at G1 stage, and prevent entering the S phase through deactivating CDKs.^31^ CDKs is also considered as a regulator of cell‐cycle progression. These findings suggest that MCM4 knockdown triggered G1 arrest through the induction of CDK inhibitor P21.

## 5 CONCLUSION

MCM4 is highly expressed in LUAD tumour samples and cells, and its upregulation is closely related to LUAD patients OS. In addition, it regulates LUAD cell proliferation, cell apoptosis and cycle in vitro, as well as tumour growth in vivo. These results provide a possible new therapy for LUAD treatment in the future.

## AUTHOR CONTRIBUTIONS

**Yue Tan:** Conceptualization (equal); investigation (equal). **Lei Ding:** Conceptualization (equal); investigation (equal). **Guiyuan Li:** Conceptualization (equal); writing – original draft (equal).

## CONFLICT OF INTEREST STATEMENT

The authors declare that they have no potential competing interests.

## Data Availability

The data used to support the findings of this study are included within the article.
